# Meta-Analysis of Permeability Literature Data Shows Possibilities and Limitations of Popular Methods

**DOI:** 10.1021/acs.molpharmaceut.4c00975

**Published:** 2025-02-20

**Authors:** Kateřina Storchmannová, Martin Balouch, Jakub Juračka, František Štěpánek, Karel Berka

**Affiliations:** † Department of Physical Chemistry, Faculty of Science, Palacký University Olomouc, 17. listopadu 12, 771 46 Olomouc, Czech Republic; ‡ Department of Chemical Engineering, University of Chemistry and Technology, Technická 3, Prague 6, 166 28 Prague, Czech Republic; § Zentiva, k.s., U. Kabelovny 130, Prague 10, 102 00 Prague, Czech Republic; ∥ Department of Computer Science, Faculty of Science, Palacký University Olomouc, 17. listopadu 12, 771 46 Olomouc, Czech Republic

**Keywords:** membrane, permeability, PAMPA, BLM, liposome, CACO-2, MDCK, PerMM, COSMOperm, MolMeDB

## Abstract

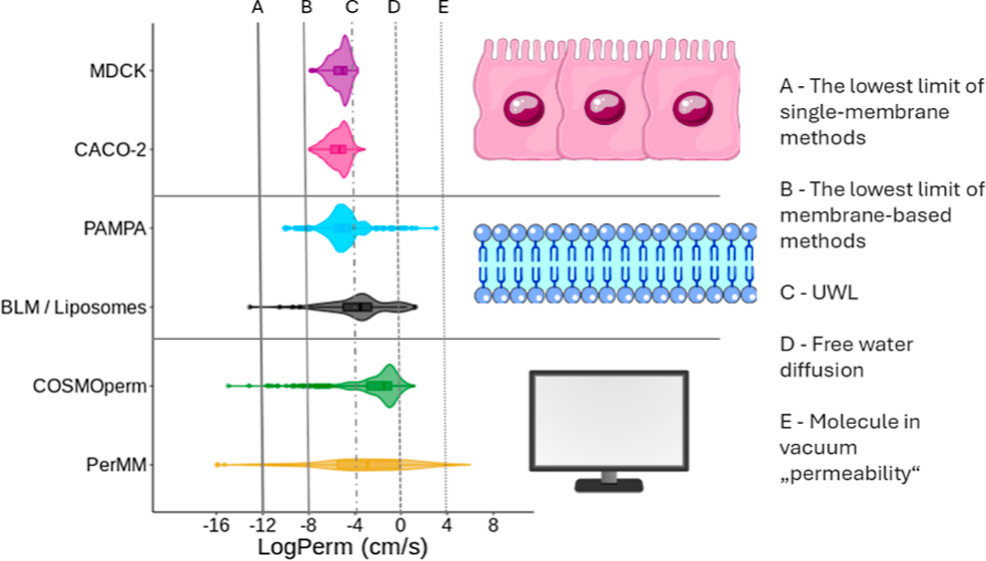

Permeability is an important molecular property in drug discovery, as it co-determines pharmacokinetics whenever a drug crosses the phospholipid bilayer, e.g., into the cell, in the gastrointestinal tract, or across the blood–brain barrier. Many methods for the determination of permeability have been developed, including cell line assays (CACO-2 and MDCK), cell-free model systems like parallel artificial membrane permeability assay (PAMPA) mimicking, e.g., gastrointestinal epithelia or the skin, as well as the black lipid membrane (BLM) and submicrometer liposomes. Furthermore, many in silico approaches have been developed for permeability prediction: meta-analysis of publicly available databases for permeability data (MolMeDB and ChEMBL) was performed to establish their usability. Four experimental and two computational methods were evaluated. It was shown that repeatability of the reported permeability measurement is not great even for the same method. For the PAMPA method, two different permeabilities are reported: intrinsic and apparent. They can vary in degrees of magnitude; thus, we suggest being extra cautious using literature data on permeability. When we compared data for the same molecules using different methods, the best agreement was between cell-based methods and between BLM and computational methods. Existence of unstirred water layer (UWL) permeability limits the data agreement between cell-based methods (and apparent PAMPA) with data that are not limited by UWL permeability (computational methods, BLM, intrinsic PAMPA). Therefore, different methods have different limitations. Cell-based methods provide results only in a small range of permeabilities (−8 to −4 in cm/s), and computational methods can predict a wider range of permeabilities beyond physical limitations, but their precision is therefore limited. BLM with liposomes can be used for both fast and slow permeating molecules, but its usage is more complicated than standard transwell techniques. To sum up, when working with in-house measured or published permeability data, we recommend caution in interpreting and combining them.

## 1 Introduction

Passive permeability is a critical molecular property studied in drug discovery because of its strong influence on the pharmacokinetics. It plays an essential role in the gastrointestinal absorption of oral drugs, penetration of the blood–brain barrier (BBB), and renal reabsorption.^1^

The permeability coefficient (cm·s^–1^) is the quantitative measure of permeability and is often presented as a decimal logarithm (log Perm). Numerous methods for their determination have been developed because of the importance of permeability coefficients in pharmaceutical research. We can, in general, divide the approaches into cell-based in vivo experimental assays, membrane-based in vitro experimental essays, and in silico approaches.

Among the oldest and best-established methods of permeability measurement are the cell-based colon carcinoma cell line permeability assay (CACO-2)^2^ and Madin–Darby Canine Kidney cells (MDCK).^3^ Permeability measurements are realized in transwell plates. Each well is divided into a donor and an acceptor compartment, separated by a membrane. In the case of CACO-2 and MDCK, the membrane consists of a cell monolayer cultured on a solid support. Despite their different origins, the CACO-2 and MDCK are composed of morphologically analogous cells and are widely used as model intestinal membranes.^4^

Apart from cell-based experiments, there are several in vitro membrane-based methods. The most often used one is the parallel artificial membrane permeability assay (PAMPA).^5^ The membrane on which PAMPA methods are based is artificially made and chosen, depending on the membrane the assay mimics. To date, many variants of PAMPA have been published. The examples include double sink PAMPA (DS-PAMPA),^6^ which mimics gastrointestinal absorption, BBB PAMPA,^7^ SkinPAMPA,^8^ or nasal-PAMPA.^9^ Another long-time-known experimental method of permeability measurement is black lipid membrane (BLM), first published by Mueller et al.^10^ In this experimental setup, membranes are prepared in the form of very thin lipid films. This method is suitable as a model of more complex natural membranes.^11,12^ The results of the BLM method are sometimes augmented by results of liposomal assays^13^ because both methods mimic permeation through simple membranes. Therefore, they are fundamentally very similar systems. Unlike CACO-2 or MDCK, which employ a monolayer of complex living cells, PAMPA and BLM methods both use simpler membranes that are unable to effect active transport (influx as well as efflux), paracellular transport, metabolism, or ion-trapping in lysosomes.^14,15^

Experimental methods for the determination of membrane permeability have been supplemented by in silico approaches, which can be divided into three main categories: molecular dynamics (MD) simulations, physics-based computational methods, and machine-learning statistical models.

MD simulations are in silico methods based on time-resolved simulations of complex systems at the atomistic level.^16^ We can derive many thermodynamic and kinetic properties of the system from the MD simulations.^16^ Thanks to the current level of computational power and MD methods, we can now study the behavior of substances on membranes even at the atomic level,^17^ but they are so far limited by the quality of membrane force fields,^18,19^ long time scales necessary for membrane permeation, and hysteresis artifacts for advanced sampling methods.^20^ Hence, the availability of these data in large quantities is still quite limited, and MD is used more to model how molecules permeate the membranes.^21,22^

The PerMM^13^ and COSMOperm^23^ are examples of physics-based calculated methods. PerMM is based on the solubility diffusion model^24^ and the positioning of proteins in membranes method.^25,26^ PerMM can also calculate the permeability coefficient across four types of membranes (DOPC, BLM, CACO-2/MDCK, and BBB).^13^ COSMOperm is a mechanistic method for the prediction of membrane permeability based on quantum chemical solubility calculations. Its basis is the calculation of the free energy profile across the membrane. In general, this approach can calculate log Perm on any membrane.^23^

Machine learning (ML) approaches are trained statistically over the existing experimental data, while the data quality and size are extremely important. The quantitative structure–activity relationship (QSAR) model is a mathematical model which identifies statistically significant correlations between the structure of molecules and their properties, such as biological activity.^27^ The structures of molecules are described by a variety of descriptors. Choosing the fitting descriptor is one of the key points during the QSAR process. The history of QSAR is long, and countless various permeability QSAR models have been developed, e.g., QSAR models for CACO-2 cell permeability,^28^ intestinal permeability,^29^ BBB^30^ and skin,^31^ etc. QSAR models were recently generalized with machine-learning (ML) models. These models are fitted to train data produced by experimental methods. Experimental methods often used for the construction of these models are CACO-2 (e.g., Wang et al.^32^ or Frelund et al.^33^) and PAMPA (e.g., Sun et al.^34^ or Gousiadou et al.^35^). Sometimes, these models are created for a given type of molecule, e.g., cell-penetrating peptides^36^ or macrocycles.^37^ As their performance can be, in principle, only as good as the original data, we do not discuss them further here.

Because of the importance of permeability and the growing volume of published data obtained by various methods, permeability data are available in well-established cheminformatics databases (e.g., PubChem^38^ or ChEMBL^39^). Nevertheless, these databases do not primarily focus on this type of data, unlike the MolMeDB database.

MolMeDB^40^ (https://molmedb.upol.cz) is a comprehensive, freely available database of membrane interaction data, including permeation for small molecules. This database stores the manually obtained data from scientific papers as well as the permeability data obtained from ChEMBL by a data mining workflow. Currently, there are more than 900,000 interactions for almost 500,000 molecules in MolMeDB. Most of the data are permeability data from 56 theoretical or experimental methods on 48 various membranes.

This paper compares and interprets the results of four experimental methods, PAMPA, CACO-2, MDCK, and BLM/liposomes, and two calculated methods, PerMM and COSMOperm, available in MolMeDB. However, in order to understand these data properly, we must first understand the methods and their constraints. Therefore, this paper has three main aims: (i) to compare methods with each other to put the log Perm quantity in a real-world context, (ii) to identify and explain the limits of the mentioned methods, and (iii) to put the log Perm quantity in a real-world context.

## 2 Methods

### 2.1 Data Sources

Data were sourced from MolMeDB and ChEMBL. The data in ChEMBL were fetched by the ChEMBL data web service. For this purpose, the KNIME^41^ semiautomatic workflow was created. This workflow fetched information about molecules (SMILES, name, and ChEMBL ID), publication (DOI), and interactions. All interactions were converted to decimal logarithms, and all units of interactions were converted to cm·s^–1^. The data mining workflow are available on WorkflowHub (https://workflowhub.eu/workflows/1169). Fetched data are available in MolMeDB. These data are labeled as ChEMBL in the Secondary reference column in MolMeDB. The content of MolMeDB was exported as a .csv file. This is possible on the Web site https://molmedb.upol.cz/stats/show_all. The source data for this meta-analysis include the method used, and membrane composition is also given. The readers can find this information in the file “prepared_MolMeDB_dataset.csv”, which is available on WorkflowHub and as a Supporting Information.

### 2.2 Analysis of Permeability Data—MolMeDB Data Selection

Data were sourced from the MolMeDB database. The PAMPA method included methods that were referred to as EPAM, EBAMP^42^ (for apparent PAMPA), and EPAMOL (for intrinsic PAMPA). The BLM/liposomes included methods EBLM and ELIP in MolMeDB (for more details, see https://molmedb.upol.cz/browse/methods).

In the cases of scatter plots (Figures 2 and S1), a mean log Perm value for molecules that have more than one log Perm value in MolMeDB was calculated. The mean values were calculated according to the rules described in the Colab notebook. Cleveland dot plots (Figure 4A,B) are created from median values of log Perm.

For greater intercomparability of data, we excluded data other than that measured or calculated on the cell membranes, generic membranes, and membranes of the intestine according to the MolMeDB classification system (for more details, see https://molmedb.upol.cz/browse/membranes). MolMeDB stores permeability coefficients (log Perm) uniformly in the logarithmic form of cm·s^–1^.

Next, the data set was narrowed down to data for small molecules (*M*_W_ ≤ 800 Da). For the computational methods (PerMM and COSMOperm), interactions where the molecules were in a neutral state were included, since only the neutral form is usually eligible to penetrate the lipid membrane.^43−45^ In the case of experimental methods (CACO-2, MDCK, PAMPA, and BLM/liposomes), only interactions for which the pH was between 7.0 and 7.5 were included. Also, experiments where pH is not explicitly stated were included because it was assumed that they were performed at standard conditions. In addition, amino acids were excluded from the final data set because of their zwitterionic character. Only data pertaining to a temperature of (20–25 °C) were included. 25 °C is the default temperature value in MolMeDB. In the case of the data from ChEMBL, the value of temperature is unknown, and for this reason, the approximately temperature of 25 °C is given in these cases. The resulting data set contained data on 5483 interactions for 4218 unique molecules (by SMILES).

Analysis was done using KNIME workflow, and figures were created by R programming language version 4.3.2^46^ or by Python 3.10.12. The Colab notebook^47^ for figures preparation is available on MolMeDB GitHub https://github.com/MolMeDB/How-Usable-Are-Published-Permeability-Data, and the KNIME workflow is available on^48^ WorkflowHub https://workflowhub.eu/workflows/1109. The UpSet plot was created by UpSetR Shiny App.^49^

In the analyzed data set, data originated from four different experimental methods, cell-based CACO-2 (1425 molecules) and MDCK (402 molecules); membrane-based PAMPA (2592 molecules) and BLM/liposomes (90 molecules); and two computational methods, PerMM (423 molecules) and COSMOperm (490 molecules).

### 2.3 Apparent and Intrinsic Permeability

In order to compare and analyze values of permeability, we must be able to distinguish between two concepts: apparent permeability and intrinsic (or molecular) permeability.

In a stirred container, the solute concentration is equalized in the bulk of the liquid. However, close to the membrane surface, molecules move by only diffusion rather than by convection. As a solute flows through the membrane, a concentration gradient builds up in close proximity to the membrane, weakening the driving force.^50^

According to eq 1, the so-called measurable apparent permeability (*P*_app_) is composed of contributions:

*P*_UWL_, *f*_neutral_, and *P*_intr_.^51^
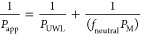
1where *P*_UWL_ is the permeation through an unstirred water layer (UWL), *f*_neutral_ is the fraction of molecule that is in a nonionized state in the donor compartment, and *P*_intr_ is the neutral molecule’s intrinsic permeability. This equation is plausible for the cases where the permeability of the ionized species is negligible. That is often valid, but there are specific examples when this assumption is not fulfilled.^52,53^ UWL is visualized as a static layer of water directly adjacent to the surface of a membrane, acting as an additional resistance to permeation. This value can vary with different stirring of donor and acceptor compartments, but for the most common experimental setting of CACO-2/MDCK or PAMPA assay, this value is around −3.9.^14^

Intrinsic permeability is often obtained from calculation based on measuring the permeability scale at different pH levels. This approach can be found in publications by Huque et al.,^54^ Avdeef et al.,^55^ or Tsinman et al.^56^ Furthermore, other implementations of this approach showed Velický et al.,^57^ where the permeabilities are measured at different hydrodynamic regimes and from that, intrinsic permeability can also be calculated.

Here, it can be noted that the apparent permeability is easily calculable from the intrinsic permeability by taking into consideration the fraction of nonionized molecules (from p*K*_a_) and the permeation rate through an UWL, which is specific to the experimental setup and can be determined from data for fast-permeating molecules. Conversely, the determination of intrinsic permeability from the apparent one is feasible only when the apparent permeability is not close to the diffusion limit. This relation limits the usefulness of published apparent permeability data for intrinsic permeability models.

## 3 Results and Discussion

### 3.1 Repeatability of the Data

First, we wanted to analyze the repeatability of the data to get the gist of the reported value variability necessary to establish error estimation. For this purpose, permeability measurements for the same molecules were considered using the same method under similar conditions (see Methods section). The most prevalent experimental method in the MolMeDB is PAMPA. We have taken the seven most studied molecules. They are listed in Table 1, along with the number of measured data points, average permeability, and standard deviation.

**Table 1:** Molecules with the Most Data Measured by the PAMPA Method^a^

	number of experimental values		average log Perm (cm/s) ± std. deviation		minimum value (cm/s)		maximum value (cm/s)	
	*P*_intr_	*P*_app_	*P*_intr_	*P*_app_	*P*_intr_	*P*_app_	*P*_intr_	*P*_app_
antipyrine	1	6	–5.09	–5.81 ± 0.23	–5.09	–6.09	–5.09	–5.54
carbamazepine	1	7	–5.33	–4.76 ± 0.67	–5.33	–5.8	–5.33	–3.89
ketoprofen	1	12	–4.32	–5.6 ± 0.35	–4.32	–6.39	–4.32	–4.9
naproxen	2	4	–2.55 ± 1.22	–5.3 ± 0.74	–3.41	–6.2	–1.69	–4.38
propranolol	3	14	–1.63 ± 1.25	–4.63 ± 0.87	–2.56	–6.68	–0.21	–3.27
theophylline	3	5	–6.17 ± 0.53	–5.8 ± 0.93	–6.77	–7.37	–5.76	–5.07
verapamil	3	6	–1.35 ± 0.4	–4.93 ± 0.25	–1.62	–5.19	–0.89	–4.61

a Data taken from MolMeDB. *P*_intr_—data for intrinsic permeabilities, *P*_app_—data for apparent permeabilities.

**Figure 1:**
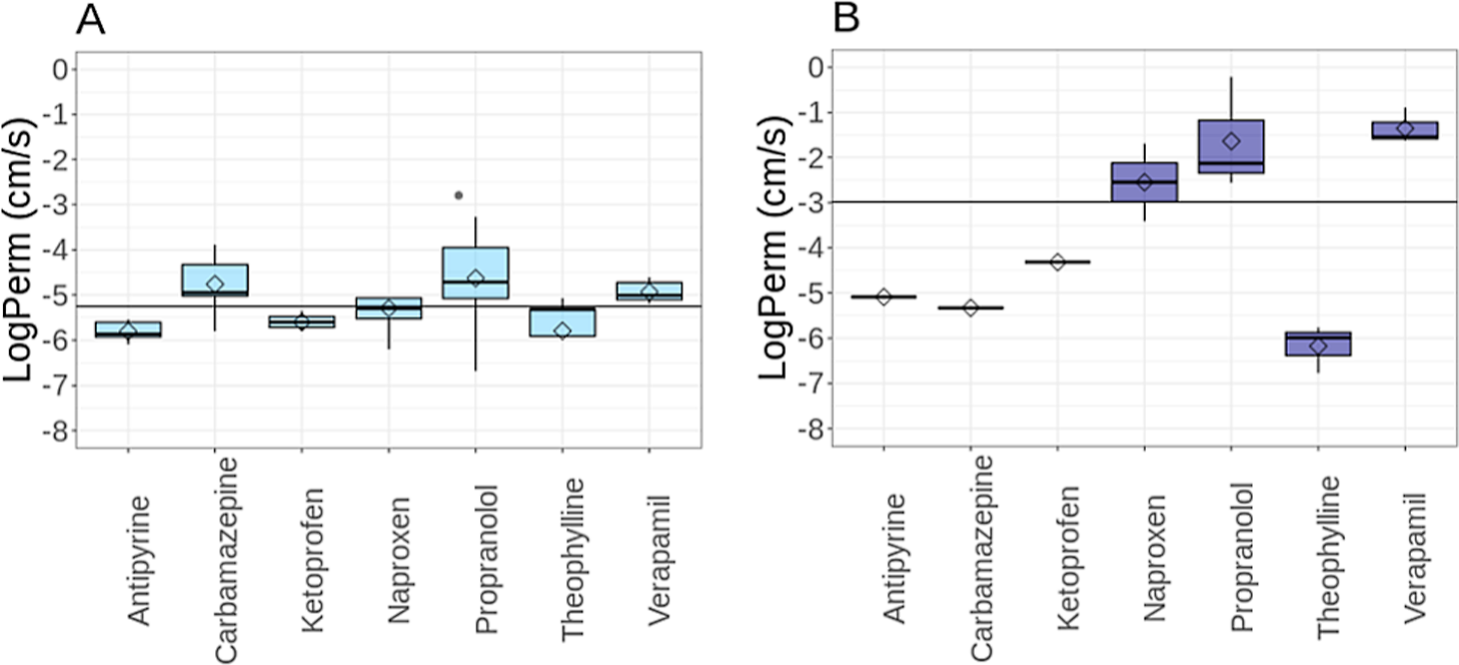
Box plot of seven molecules with the most data measured by the PAMPA method (in MolMeDB). A—apparent permeability (*P*_app_), B—intrinsic permeability (*P*_intr_). Rhombuses represent mean values of permeability, and the line corresponds to the median value of the data set. Dots represent outliers.

When visualizing the data from these seven molecules measured by PAMPA (Figure 1), we see several different behaviors of molecules that can be discerned between intrinsic and apparent permeabilities.

First, there are molecules with very low variance between measured values, such as antipyrine or ketoprofen, within 0.5 log unit. Further molecules, such as carbazepine or theophylline, have reasonable variances within 1 log unit. Table 1 shows several molecules with variance within 2 log units; hence, individual permeability measurements differ by more than 2 orders of magnitude. However, these large errors can be explained by the mixing of apparent and intrinsic permeability, which Figure 1 shows to be several log units different for those molecules.

Unfortunately, the information on whether intrinsic or apparent permeability is reported is not always properly described in the literature or databases (e.g., we had to implement PAMPA *P*_intr_ into MolMeDB upon preparation of this manuscript). The lack of distinction between apparent and intrinsic permeability can lead to complications, especially when data from multiple papers using the PAMPA method.

A similar lack of information affects cell-based methods. There is crucial information about the direction of measurement or membrane transport of proteins. There are two possible directions, either from apical to basolateral (from A to B) or from basolateral to apical (from B to A). The difference of permeabilities in direction can be large, e.g., in Colabufo et al.^58^ The direction affects the value of measured permeability because these cells have an asymmetrical expression of pharmacologically relevant proteins that influence molecular transport.^59^ These proteins are (i) efflux transporters, e.g., *P*-glycoprotein (MDR1, ABCB1), MRP2 (ABCC2) or BCRP (ABCG2), and (ii) uptake proteins, e.g., OCT2 (SLC22A2), OATP2B1 (SLCO2B1), or PEPT1 (SLC15A1).^59,60^ In the case of these measurements, not only the direction in which the permeability is measured but also the presence of transporter inhibitors plays a role.^33,59^ The inhibitors increase the permeability of substrates of efflux transporters.^33^ Gene knockout techniques can also alter the expression of these transporters in the cells.^60^ The chemical composition of the donor compartment solution is a further aspect that has strong influence to results of permeability assays as bovine serine albumin is a protein that can improve permeability.^61^ In addition, donor compartment solution can simulate fasted and fed states. For this reason, there are used simulated intestinal fluids.^61^ In previous years, there were published papers which are focused on other experimental aspects related to cell-based permeability methods, e.g., by Hubatsch et al.^62^ Unfortunately, this information is often not sufficiently reported together with permeability data in publicly available databases.

### 3.2 Comparison of Methods

In this analysis, we studied the correlation between each pair of permeability methods to see how they can be supplemented with each other if needed. A mean log Perm value for each molecule and method was calculated, and for molecules that were measured and calculated by at least two methods, these data were compared. The comparison of all possible pairs of methods is available in Supporting Information (Figure S1). Here, we discuss the most prominent and illustrious examples.

**Figure 2:**
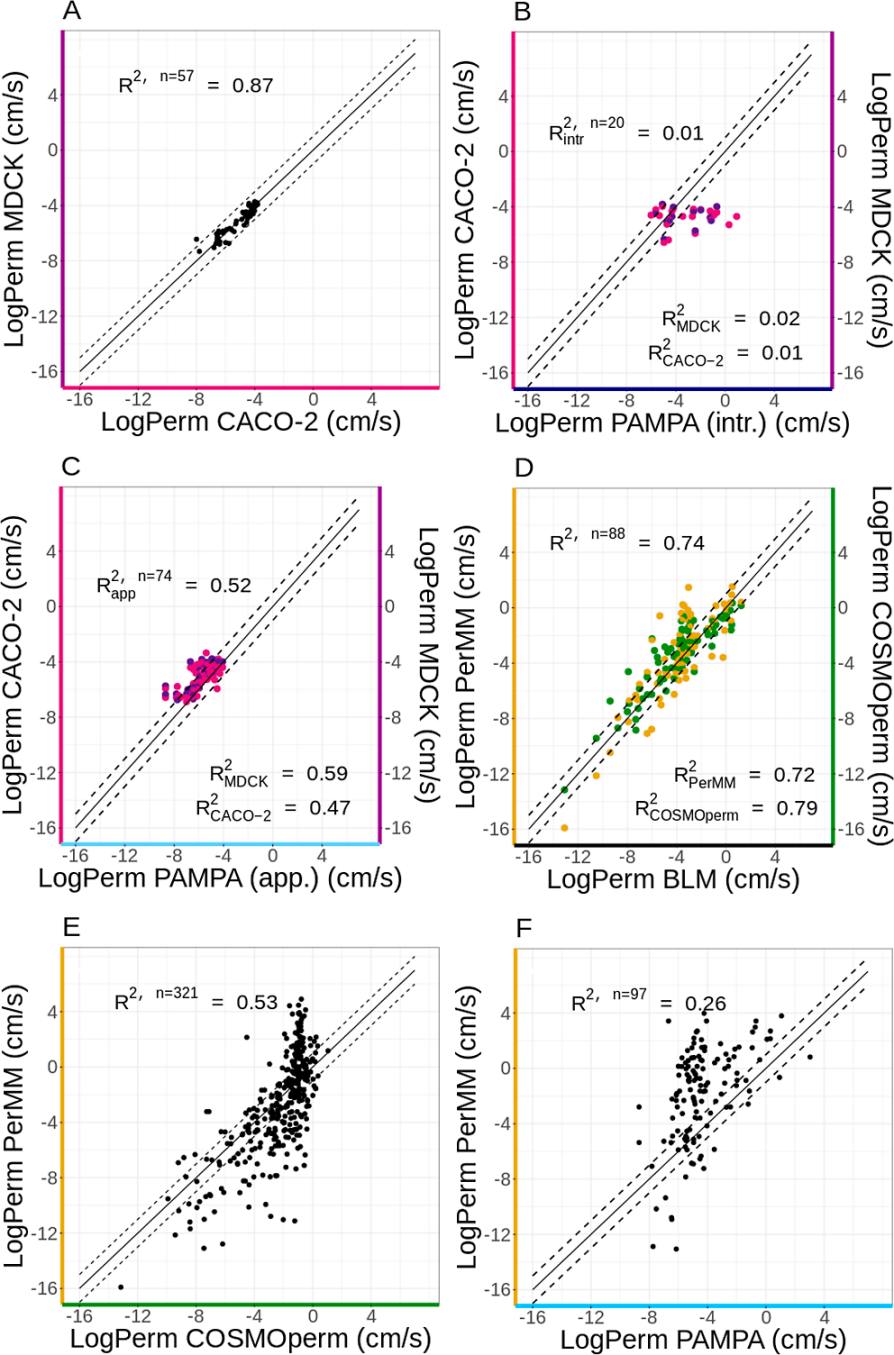
Mean log Perm values of molecules that are present in overlaps between (A) CACO-2 and MDCK data sets; (B) CACO-2 and MDCK and intrinsic PAMPA data sets; (C) CACO-2 and MDCK and apparent PAMPA data sets; (D) COSMOperm and PerMM and BLM/liposomes data sets; (E) PerMM and COSMOperm data sets; (F) PerMM and PAMPA data sets (apparent and intrinsic permeabilities). The solid line represents the parity of permeation values, and the dashed lines represent a log Perm difference of ±1 between methods. All data are displayed in a log of cm·s–1. *N* is the number of unique molecules in overlap, and *R*^2^ is the coefficient of determination.

The first finding is the strong correlation (*R*^2^ = 0.87) between CACO-2 and MDCK (Figure 2A). This correlation is expected because both methods are cell-based methods, and their strong correlation was described by Irvine et al.^3^

From the previous section, we know the PAMPA data set contains a mixture of apparent and intrinsic permeabilities that need to be differentiated if the data are to be compared. Figure 2B shows the correlation among CACO-2 and MDCK vs intrinsic PAMPA. This correlation is very weak (*R*^2^ = 0.01) because, in contrast to PAMPA, the CACO-2 and MDCK methods typically provide apparent permeability, as these media are usually less disturbed with experimental conditions using different pH levels or mixing. On the other hand, Figure 2C shows the correlation (*R*^2^ = 0.52) between CACO-2 and MDCK vs apparent PAMPA. This correlation is unsurprisingly stronger because the PAMPA apparent permeabilities correlate well with CACO-2 and MDCK data. This observation is consistent with the literature because the correlation among these methods was already described in the literature (PAMPA and CACO-2 were described by Zhu et al.,^63^ MDCK and PAMPA were published by von Von Richter et al.^64^). However, in addition to the membrane, cell-based methods CACO-2 and MDCK also have membrane transport proteins, which can influence the transport of molecules through the membrane and thus lower the correlation. However, the correlation between CACO-2 and PAMPA and MDCK and PAMPA indicates passive diffusion as a dominant transport mechanism in the case of both cell monolayers.^11,65^ A good correlation between CACO-2 and MDCK vs apparent PAMPA can indicate a smaller role of membrane transporters in this data set.

Figure 2D shows a strong correlation (*R*^2^ = 0.74) between the calculated methods (PerMM and COSMOperm) and BLM/liposomes (abbreviated “BLM” in the figure). Since both of these methods use simple models of membranes, they certainly have the closest relevance to physics-based computational methods.

The authors of both calculated methods validated these methods against BLM experimental data.^13,23^ Bittermann et al.^66^ reported that COSMOperm has RMSE = 0.62 log units for neutral molecules and RMSE = 0.7 log units for ions. The PerMM method has RMSE = 1.15 log unit by Lomize et al.^13^ Therefore, it is no surprise that these methods correlate strongly. In addition, highly similar BLM data sets were used for validation, and this validation data comprises the majority of the BLM data in the MolMeDB database. We also see that the values are wide-ranging because BLM, COSMOperm, and PerMM are methods that are not constrained by diffusion limits and provide intrinsic permeabilities.

Figure 2E shows a correlation between calculated (PerMM and COSMOperm) (*R*^2^ = 0.52). This correlation is not surprising, given what has been said about these methods above. Both methods are unlimited by diffusion limit and can predict intrinsic permeabilities.

Figure 2F shows an example of the weak correlation between diffusion-limited method (PAMPA) and unlimited method (PerMM). More examples can be found in Supporting Information [Figure 1 S (H and O)]. As we can clearly see, PAMPA data are located in the range of values (approximately from −8 to 4 log units), but the PerMM method is in the wider range of values (approximately from −16 to 4). Differences between the log Perm values from PerMM and PAMPA can be huge (several log units). However, the PerMM method was successfully evaluated against DS-PAMPA method (DS-PAMPA: *R*^2^ = 0.75, RMSE = 1.59 log unit) by Lomize et al. in the original paper.^13^ Our correlation is weaker (*R*^2^ = 0.26) than Lomize’s because our data set contains apparent permeabilities and intrinsic permeabilities, whereas Lomize used only intrinsic permeabilities from one source.

Apart from the correlation, it is often more useful to calculate the mean absolute error (MAE) for each comparison (Table 2). It shows that the closest pair of methods are both cell-based methods (CACO-2 and MDCK) followed by their pairs with their membrane-based counterpart sharing similar range — apparent PAMPA. The error between BLM and both computational methods is comparable to the MAE in between them, but their similarity to cell-based methods is weak with the largest error. As a negative control, we have tried mean predictor, i.e., we calculated MAE of each method toward the mean average value calculated on its data set. This value serves as a negative control for the fit to that data set. If the MAE value for pair is lower or at least similar than MAE to mean predictor, then it can be combined. This comparison has shown that we can combine both cell-based methods (CACO-2 and MDCK) together with apparent PAMPA. Similarly, both computed physics-based methods (COSMOperm and PerMM) can be used to predict membrane-based BLM method and to some extent also to intrinsic PAMPA.

**Table 2:**
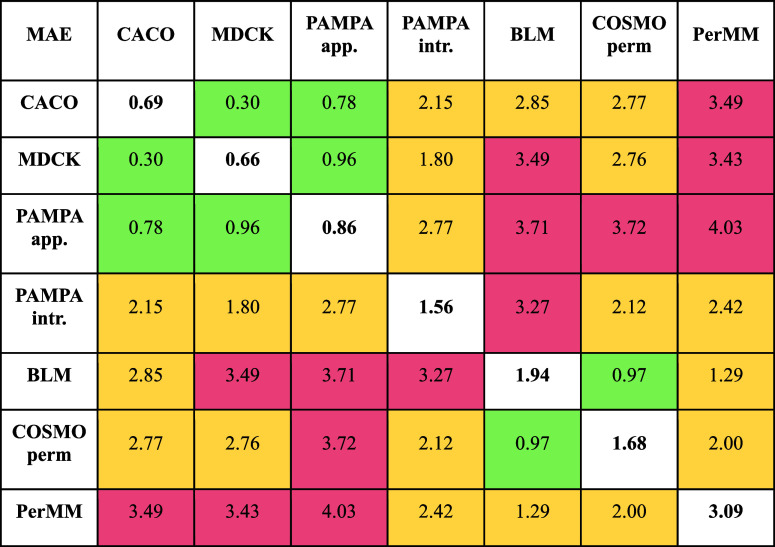
MAE for Each Pair of Methods^a^

a Colors define indicate the ranges—green has MAE range <1 log units, yellow has MAE range between 1 and 3 log units, and red has MAE range >3 log units. Diagonal shows MAE of mean predictor, i.e., toward mean average value of each dataset. This value serves as a negative control for the fit to that dataset. If the MAE value for pair is lower or at least similar than MAE to mean predictor, then the datasets can be combined.

### 3.3 Overlaps of the Methods

All of the above-mentioned methods are well-known, and the permeabilities of small molecules determined by these methods have been published in many publications.

**Figure 3:**
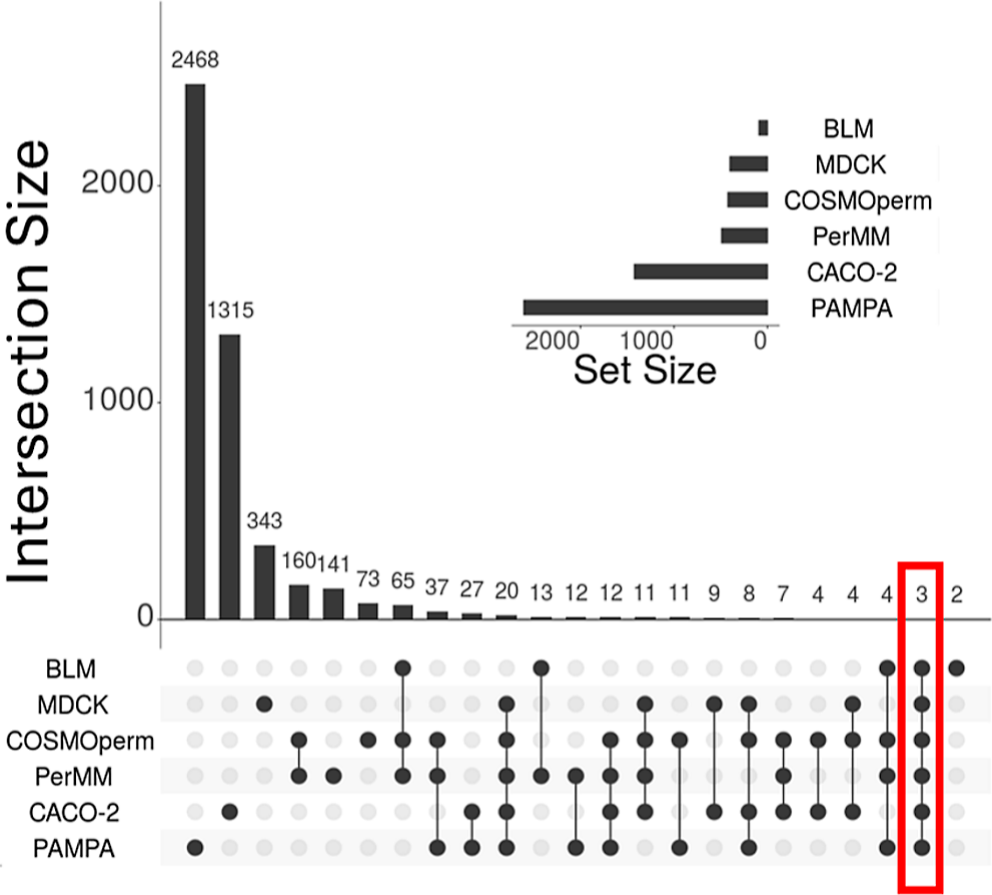
Upset plot of six methods (created with UpSetR Shiny App). Columns in the graph represent a number of molecules that were measured by the combination of methods shown by black circles only. The first column shows molecules that were measured by PAMPA only, and the last column shows molecules that were measured by PAMPA, calculated by PerMM but which are not present in any other data set. This figure includes only intersections with more than one molecule. The overlap among all six methods is highlighted by red rectangle.

The UpSet plot (Figure 3) shows overlap among all six methods by molecules (MolMeDB IDs). The biggest overlap is between both calculated methods (160 molecules); the second biggest one is the overlap among PerMM, COSMOperm, and BLM (65 molecules), and all other overlaps are much smaller.

**Figure 4:**
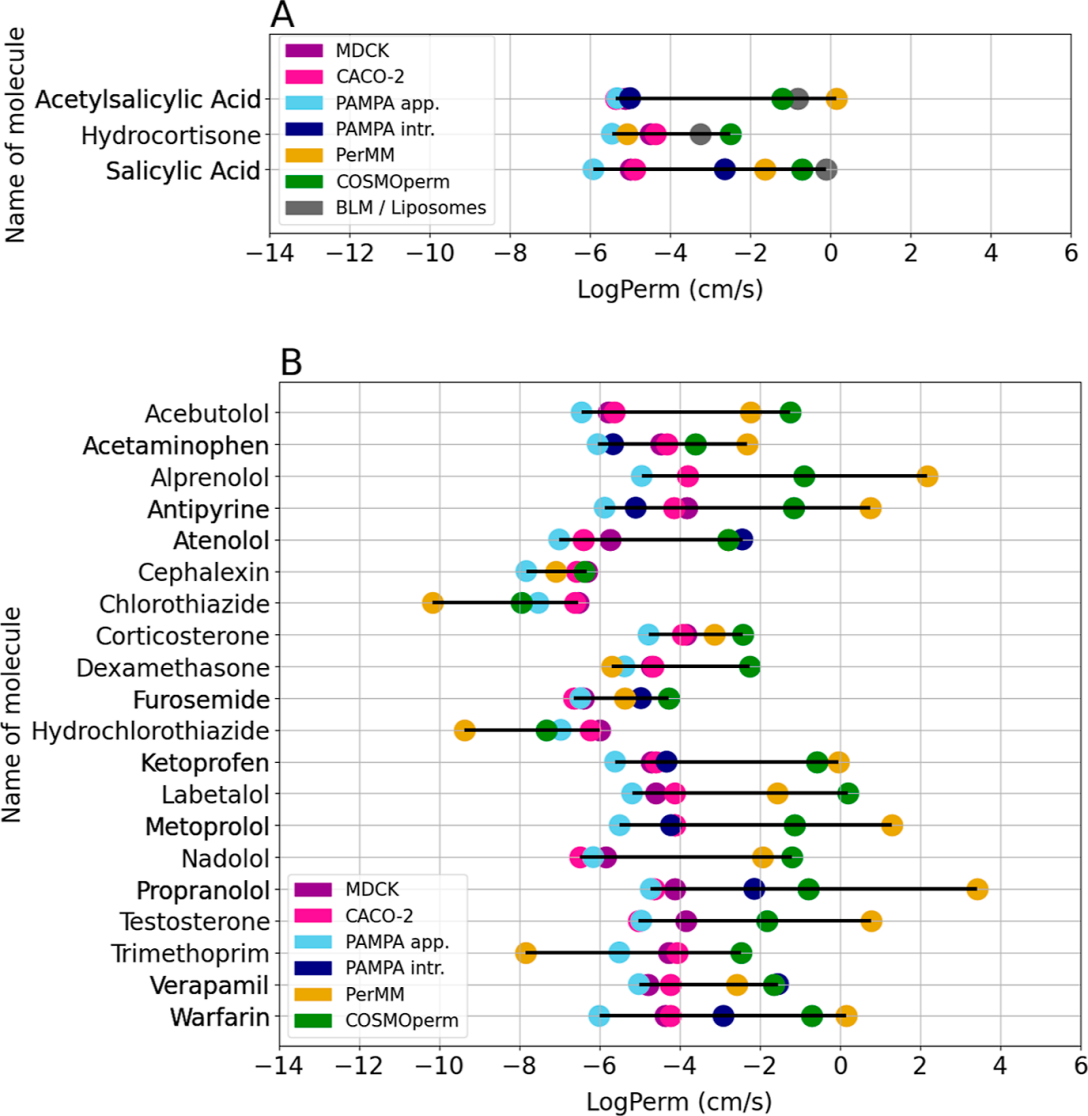
Cleveland dot plot of median permeability coefficients for molecules in the overlap of MDCK, CACO-2, PAMPA, BLM/Liposomes, COSMOperm, and PerMM data sets.

Overlap among all six methods contains three well-known drugs (hydrocortisone, salicylic acid, and acetylsalicylic acid). Figure 4A shows the Cleveland dot plot of log Perm median values from individual methods for each of these molecules. Figure 4B shows median permeability values for molecules that are present in all data sets except BLM due to the low number of molecules in the BLM/liposome data set. The data shown in Figure 4 underline the phenomena from the previous chapter. MDCK, CACO-2, and apparent PAMPA (purple, pink, and light blue points) often give very similar results. In the case of atenolol, verapamil, or warfarin, the difference between apparent PAMPA (light blue points) and intrinsic PAMPA (navy blue points) can be huge. Also, the figure demonstrates the variability of log Perm values from all calculated methods (green and yellow points).

### 3.4 Limits of Permeability of the Compared Methods

**Figure 5:**
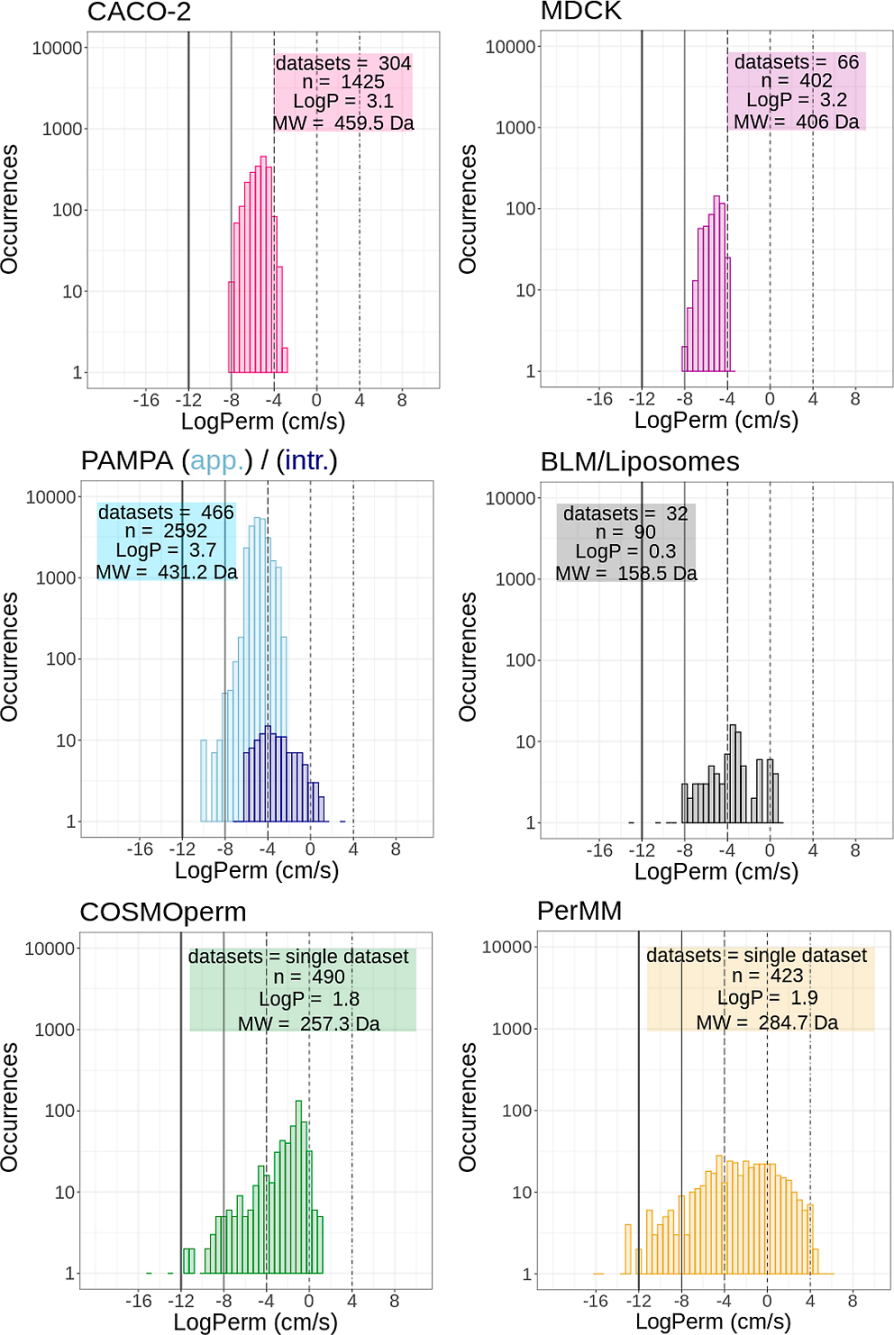
Distribution of selected log Perm values (uncharged molecules, 25 °C, smaller than 800 Da) from MolMeDB database according to selected methods. Data sets—number of unique data sets (by primary reference), log *P*—mean log *P* of unique molecules, *M*_W_—mean molecular weight of unique molecules, *n*—number of unique molecules (by SMILES) in combined data sets. All data are displayed in a log of cm/s, bin size = 0.5. The vertical lines emphasize values −12, −8, −4, 0, and +4, as discussed above. Occurrences are in log scale.

Distributions of values for individual methods have shown that each method has some limits of reported permeability that differ. Figure 5 shows that almost all CACO-2 and MDCK experimental values fall in a fairly narrow range from −8 to −4 log units. The lower limit is probably due to the duration of the experiments. It is hard to measure slower permeation under experimental conditions within ambient times. The upper limit (around −3.9) corresponds to the diffusion limit through an UWL,^14^ as explained above. Here, it can be noted that at least 40% of molecules studied using CACO-2 or MDCK assays were close to this diffusion limit. Therefore, a significant amount of measured permeability is, in fact, diffusion of the UWL, and their intrinsic permeability is thus anywhere between −4 and 4. However, for example, Stenberg et al. et al. published CACO-2 data where they tried to reduce the effect of UWL on permeability by using two stirring rates (100 and 500 rpm).^67^ Detailed analysis of the data from this paper shows that the effect of stirring on UWL increases permeability by about −0.8 to +0.2 log unit, and it is most prominent for fast permeating molecules with high log *P* (Table S1). As a result, lipophilic molecules are generally more affected by UWL thickness than polar molecules.

Data from the PAMPA experimental assay (Figure 5 PAMPA) have a visible peak around log Perm = −4, though higher permeation rates were also measured. This phenomenon is again caused by the above-mentioned mixture of apparent and intrinsic PAMPA permeabilities in the literature. Some publications (e.g., refs (65, 68, and 69)) report the apparent permeability as a direct experimental value of permeation, which is related to CACO-2 permeability. On the other hand, other publications of PAMPA permeation data (e.g., refs (54,55)) report the intrinsic permeability, thus mixing permeability values. As can be seen from the figure, the apparent permeability (light blue) peaks between values −8 and −4, while the intrinsic permeability (navy blue) is shifted to higher values. However, some values of apparent permeability are over the −4 threshold. This phenomenon can be explained by, e.g., effort to reduce the UWL layer in permeability assay by increasing of stirring speed.^70^ Fujikawa et al. present, e.g., for desipramine log Perm = −4.77 (0 rpm), log Perm = −4.00 (200 rpm), and log Perm = −3.81 (250 rpm).^69^

Permeabilities measured by the BLM/liposome method are typically higher than −4, likely due to the relatively large membrane area in liposomal systems. Although the BLM/liposome method also provides apparent permeabilities, the UWL, in this case, is significantly smaller than that of CACO-2, MDCK or PAMPA, and its contribution is practically negligible. The lowest experimentally measured permeability value is −13.1 log units for saccharose from Brunner et al.^71^ Its authors say that any value lower than −10 log unit is hardly measurable.

With the computational approaches PerMM and COSMOperm (Figure 5 PerMM and COSMOperm), we observe a broad distribution of permeation rates from very slow permeation (log Perm ≤−8 cm·s^–1^) to very fast permeation (log Perm ≥−4 cm·s^–1^). This is typical for calculated methods because they have no experimental limits. We can compare the result from the experimental method with the result from the calculation, but only up to the limits of the experimental methods. Beyond these limits, there is no possibility of comparison. The permeabilities obtained by these calculated methods can be categorized as intrinsic permeabilities.

It must also be mentioned that the ratio of charge components is often unknown in the case of the apparent permeability of ionizable molecules, whereas intrinsic permeabilities include only neutral forms of molecules. We must take this fact into account when comparing different permeability methods, although the fraction of the neutral form is often not an easily obtainable value.

In addition, it is interesting to mention differences in averages of the octanol/water partition coefficient (log *P*) and molecular weight (*M*_W_) for molecules analyzed by different methods.

CACO-2, MDCK, and PAMPA have considerably higher averages of both values for molecules than those of the other three methods. This is probably caused by the fact that most of the permeating molecules measured in these assays in scientific publications are drug-like molecules, and the molecular weight of around 400 Da and log *P* around 3 corresponds to a typical drug candidate.

BLM/liposome methods studied molecules with typically lower *M*_W_ and log *P* (*M*_W_ = 158.5 Da; log *P* = 0.3). This is probably because these methods are not commonly used for extensive drug candidate molecule assays; most measurements are performed for small molecules (e.g., benzoic acid). BLM/liposome data are often used as training/validation of different computational methods since they have a range larger than the −8 and −4 thresholds that are hard to overcome for other experimental methods as liposomes have large surface/volume ratio enabling extremely slow permeation, and BLM enables quick stirring to overcome UWL.

Computational methods (COSMOPerm and PerMM) can predict the permeabilities of a wide range of molecules. Their average log *P* of around 1.8 and *M*_W_ of around 260–280 Da is lower than for experimental methods. This has at least two co-occurring reasons. First, using computational methods, it is possible to calculate small molecules (oxygen, water, and carbon dioxide) that are commonly not measured in permeation assays. Second, the calculation difficulty scales with molecular weight, and therefore, it is more expensive to calculate molecules with larger *M*_W_.

### 3.5 Interpreting Permeability Coefficients Real-World Time Scales

**Figure 6:**
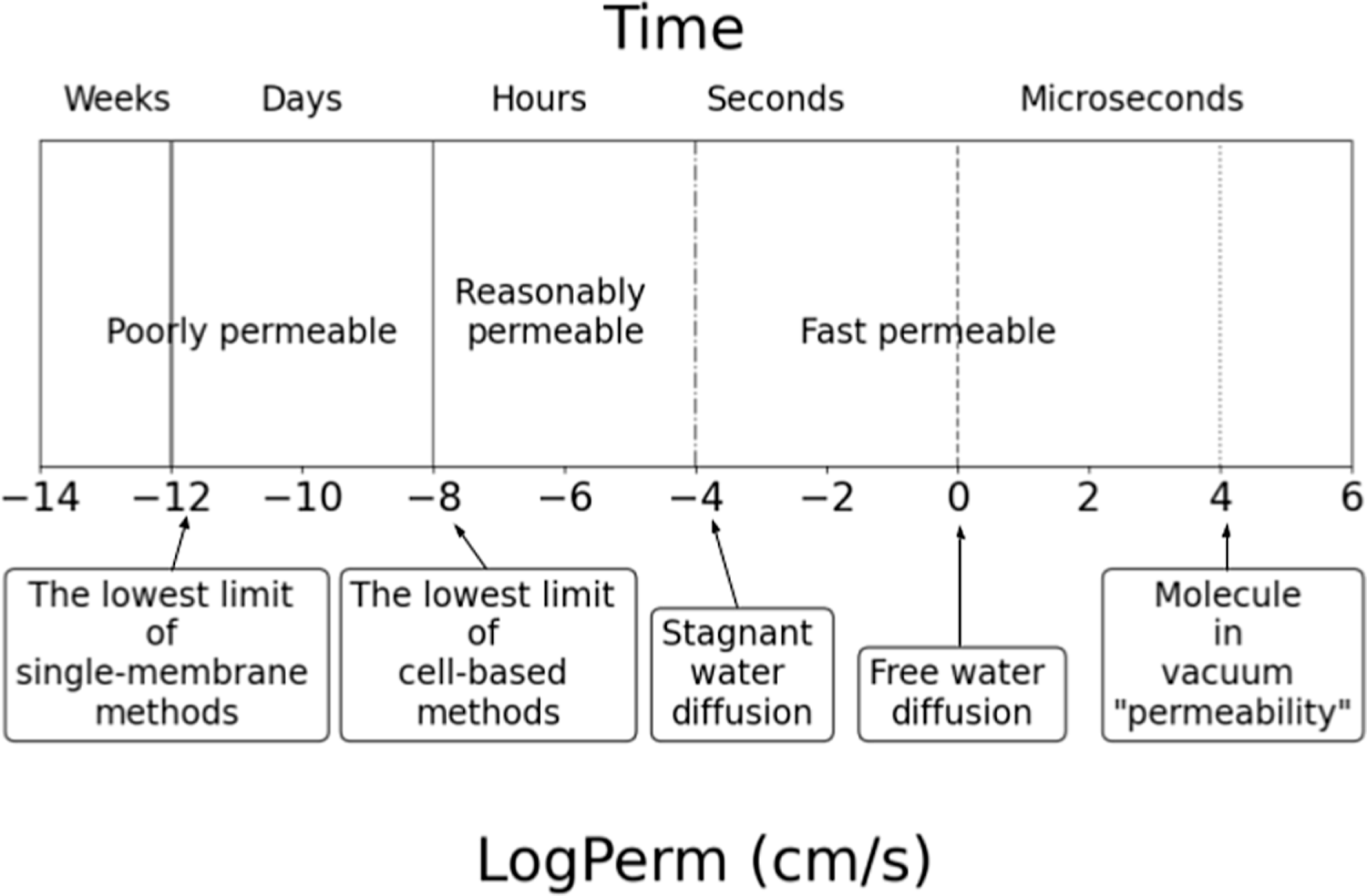
Illustration of permeability coefficients in the context of time scale.

For the interpretation of the limits we observed within the previous sections, we have designed several simplified boundaries as a multiplication of 4 log units, which can help to explain the limitations of permeability coefficients in real world examples of the time scale for permeation events (Figure 6).

The lowest experimental value of log Perm is −13.1 log units in our data set (Figure 3 BLM/liposomes).^71^ Hence, the first line of log Perm around −12 log units corresponds to the lowest permeation rates still measurable by single-membrane experimental methods. It is close to the practical limit of the slowest passive permeation that still results in a biologically feasible amount of, e.g., highly toxic compounds permeating over a physiologically relevant amount of time. For a permeation area equal to that of an entire human intestine (30 m^2^)^72^ and 0.1 L as a volume of the intestinal fluid,^73^ only 0.25% of a permeant will have permeated in 10 days at such a rate with log Perm = −12 log units.

The second line is log Perm around −8 log units. This area corresponds to the lowest limit of cell-based methods (e.g., CACO-2 or MDCK) as well as PAMPA. With the typical measurement setup for these measurements (donor volume 0.5 mL and permeation area 1.4 cm^2^),^62^ the compound with log Perm = −8 log units will permeate approximately 2% of the permeant in 10 days. The same limit is observable in, e.g., Deur et al.^74^ for CACO-2, Chiba et al.^75^ for MDCK, or Flaten et al.^76^ for PAMPA.

The third important value of log Perm is −4 log units. This represents the value of −3.9 log unit that corresponds to the effect of an UWL, as we already discussed earlier in the previous section. Hence, the typical permeability coefficient values will fall in between log Perm −8 and −4 log units.

The fourth line of log Perm around 0 log units relates to an unrestrained diffusion in water and represents the maximum permeability measurable by experimental methods in water medium. Such log Perm values correspond to the permeation of molecules through a water slice of similar thickness to the membrane, where there is no energy barrier for permeation and the diffusion coefficient is equal to the water self-diffusion coefficient. Then, the homogeneous solubility model for the permeation coefficient is valid
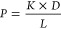
2where *K* is the partition coefficient between the membrane and water phase (considered equal to 1, if there is no extra partitioning into the slice due to no energy barrier), *D* is the diffusion coefficient (3 × 10^–9^ m^2^/s for water self-diffusion, taken from ref (77)), and *L* is the thickness of the membrane (the thinnest experimentally possible membrane with a thickness around 4 nm). Then, *P* is 75 cm/s and log Perm is +1.9 log units. Since drugs are bigger molecules than water, they usually have a diffusion constant lower by an order of magnitude or more (e.g., ibuprofen has *D* = 5.5 × 10^–10^ m^2^/s),^78^ and we have set this simplified limit to 0 log unit.

The rightmost highlighted value of log Perm is around +4 log units. This value of log Perm presents the theoretical upper limit of permeability, describing an unrestrained molecule traveling through a vacuum (together with a spherical chicken from classical physics joke). This was calculated using the formula for the root-mean-square velocity of a gas molecule (eq 3)
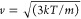
3where *k* is the Boltzmann constant, *T* is the temperature (K), and *m* is the mass of the molecule. If we assume the mass of the molecule as 200 g·mol^–1^ and a temperature of 37 °C, we get a velocity of 6000 m·s^–1^, which can be considered as a gas permeability limit (log Perm = +5.8 log units of cm·s^–1^) but only in the case of a negligible thickness of the membrane, area of permeation equal to the projection is of the molecule itself, and maximum possible concentration difference. This value is purely hypothetical and does not correspond to the biomembrane permeability in real liquid conditions. It is only stated here as the absolute upper limit of the permeability. Even the 60 times smaller value of permeability (log Perm = +4 log units of cm·s^–1^) is still purely hypothetical and impossible to obtain in biomembrane permeation; thus, this limit is used in graphs and discussion below due to the 4 orders of magnitude difference between all other limits.

## 4 Conclusions

In summary, we meta-analyzed a large amount of permeability data from the freely available databases MolMeDB and ChEMBL gathered from the literature. Permeability is, among other things, the basis of classifying drug substances into the Biopharmaceutics Classification System (BCS)^79^ and this classification forms the basis of product formulation and regulatory approval strategy decisions; hence, it is important to have reliable data for permeability. Moreover, permeability as an important pharmacological property is of interest to many researchers who try to create ML algorithms for its prediction. The variability between individual measurements, even for the same methods, has shown that efforts should be made to develop robust methods that would enable consistent interlaboratory values to be measured and stored in FAIR manner, e.g., in MolMeDB database.

The analysis of individual methods showed their limitations. Meta-analysis in-between the data sets has shown that cell-based methods such as CACO-2 and MDCK are comparable with apparent PAMPA, but all these methods correlate less with calculated physics-based methods (COSMOperm and PerMM) and with single membrane-based BLM/liposomes or intrinsic PAMPA, which are based on molecular permeabilities. This needs to be considered when permeability data from different methods are compared with or used in machine-learning approaches. Finally, we have devised a scale with five significant permeability values as a multiplier of 4 log units of cm·s^–1^ to help a comprehensive understanding of the permeability data within their physical context.

From the point of view of user or publisher of permeability data, we strongly suggest:

First, clearly state/read if the reported permeability data are apparent or anyhow calculated as an intrinsic. Second, to interpret data in limitations of used methods, for example, for apparent permeability around −4 cm/s, assume that this is strongly limited by UWL permeability. Finally, keep in mind data interpretation (apparent vs intrinsic) and also uncertainty, which is often not reported, especially for computational methods, which provide single permeability value, but in fact can be sensitive to charge state or conformation.

## Supporting Information Available

Comparison of mean permeation coefficients of individual molecules in overlaps between data sets, intervals and medians of log Perm values from individual methods including variability of reported data, recalculated apparent permeabilities with and without UWL and with their difference, and uptake ratios taken from Stenberg et al.^67^ and log *P* taken from the MolMeDB database (PDF)
Datafile with exported data set from MolMeDB used in the paper (XLS)
The file can be also accessed together with KNIME workflow at https://workflowhub.eu/workflows/1109.
