# Supplementary material for: Meta-Analysis of Permeability Literature Data Shows Possibilities and Limitations of Popular Methods
Source: Mol Pharm. 2025 Feb 20;22(3):1293–304. doi: 10.1021/acs.molpharmaceut.4c00975 (PMC11881145; doi:10.1021/acs.molpharmaceut.4c00975)
Supplement: Supplementary file 1 — mp4c00975_si_001.pdf [file mp4c00975_si_001.pdf]

Supporting Information

For

Meta-analysis of permeability literature data shows possibilities and limitations of popular methods†

Kateřina Storchmannová[1], Martin Balouch[2,3], Jakub Juračka[1], František Štěpánek[2], and Karel Berka*[1]

Figure S1: Comparison of mean permeation coefficients of individual molecules in overlaps between datasets:

A: CACO-2 and COSMOperm,

B: PAMPA and COSMOperm,

C: PAMPA and CACO-2,

D: PAMPA and BLM,

E: CACO-2 and BLM,

F: COSMOperm and BLM,

G: COSMOperm and PerMM,

H: CACO-2 and PerMM,

I: PAMPA and PerMM,

J: PerMM and BLM,

K: MDCK and BLM,

L: MDCK and CACO-2,

M: MDCK and COSMOperm,

N: MDCK and PAMPA,

O: PerMM and MDCK.

Each point represents one molecule with permeation coefficients determined by both respective methods. The solid line represents equal values; dashed lines represent a logPerm difference of ±1 log unit between methods. All data is displayed in log of cm/s, n - number of unique molecules in overlap, R^2 - coefficient of determination (in case of the PAMPA method, this value includes intrinsic and apparent permeability).

[1] *Department of Physical Chemistry, Faculty of Science, Palacký University Olomouc, 17. listopadu 12, 771 46 Olomouc, Czech Republic*

**Email: karel.berka@upol.cz*

[2]*Department of Chemical Engineering, University of Chemistry and Technology, Prague, Technická 3, 166 28 Prague 6, Czech Republic.*

[3]*Zentiva k.s., U Kabelovny 130, 102 00 Prague 10, Czech Republic*

† Electronic Supplementary Information (ESI) available: [details of any supplementary information available should be included here]. See DOI: 00.0000/00000000.

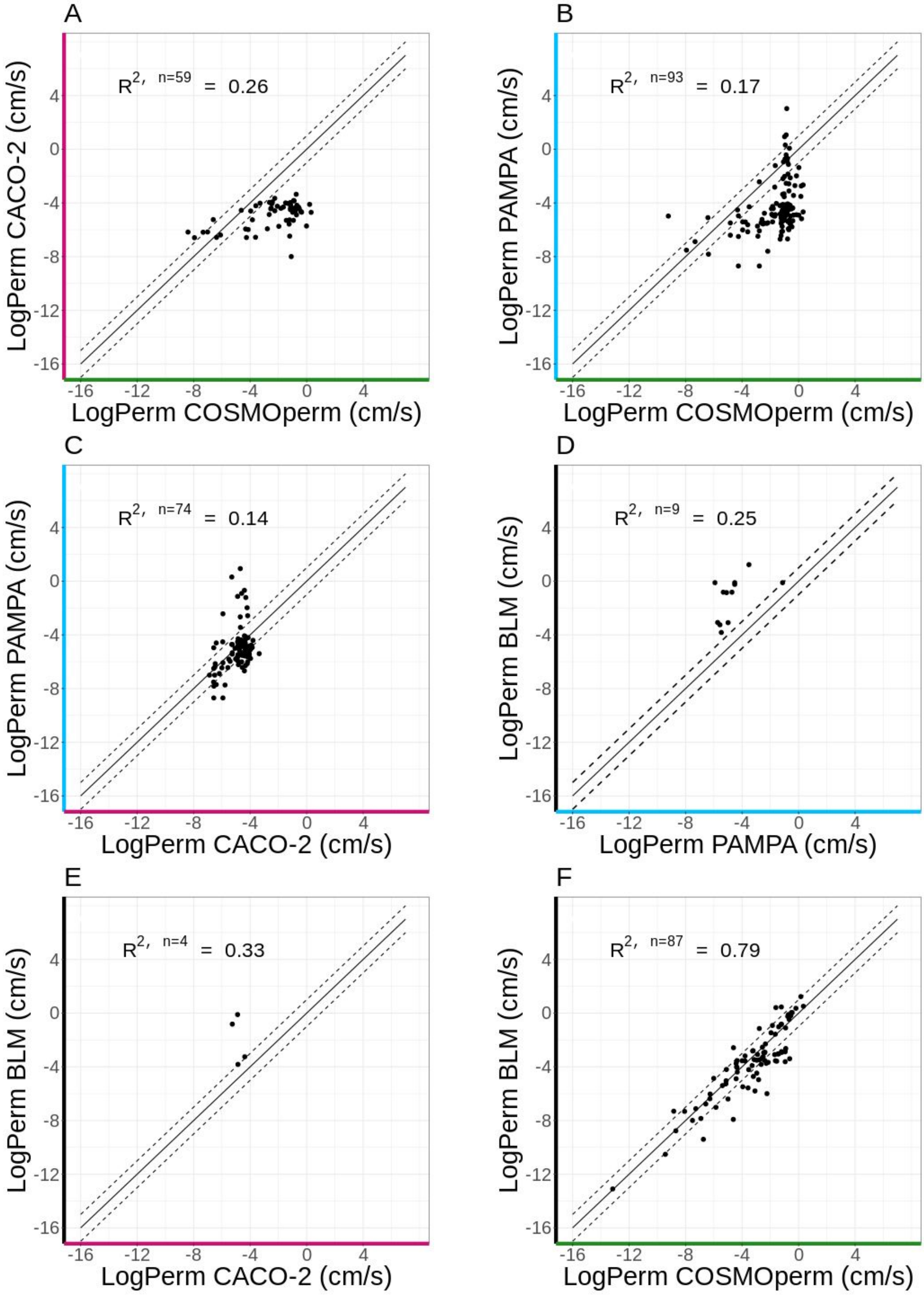

A
R2, n=59 = 0.26
LogPerm CACO-2 (cm/s)
LogPerm COSMOperm (cm/s)
B
R2, n=93 = 0.17
LogPerm PAMPA (cm/s)
LogPerm COSMOperm (cm/s)
C
R2, n=74 = 0.14
LogPerm PAMPA (cm/s)
LogPerm CACO-2 (cm/s)
D
R2, n=9 = 0.25
LogPerm BLM (cm/s)
LogPerm PAMPA (cm/s)
E
R2, n=4 = 0.33
LogPerm BLM (cm/s)
LogPerm CACO-2 (cm/s)
F
R2, n=87 = 0.79
LogPerm BLM (cm/s)
LogPerm COSMOperm (cm/s)
4
0
-4
-8
-12
-16
-16
-12
-8
-4
0
4

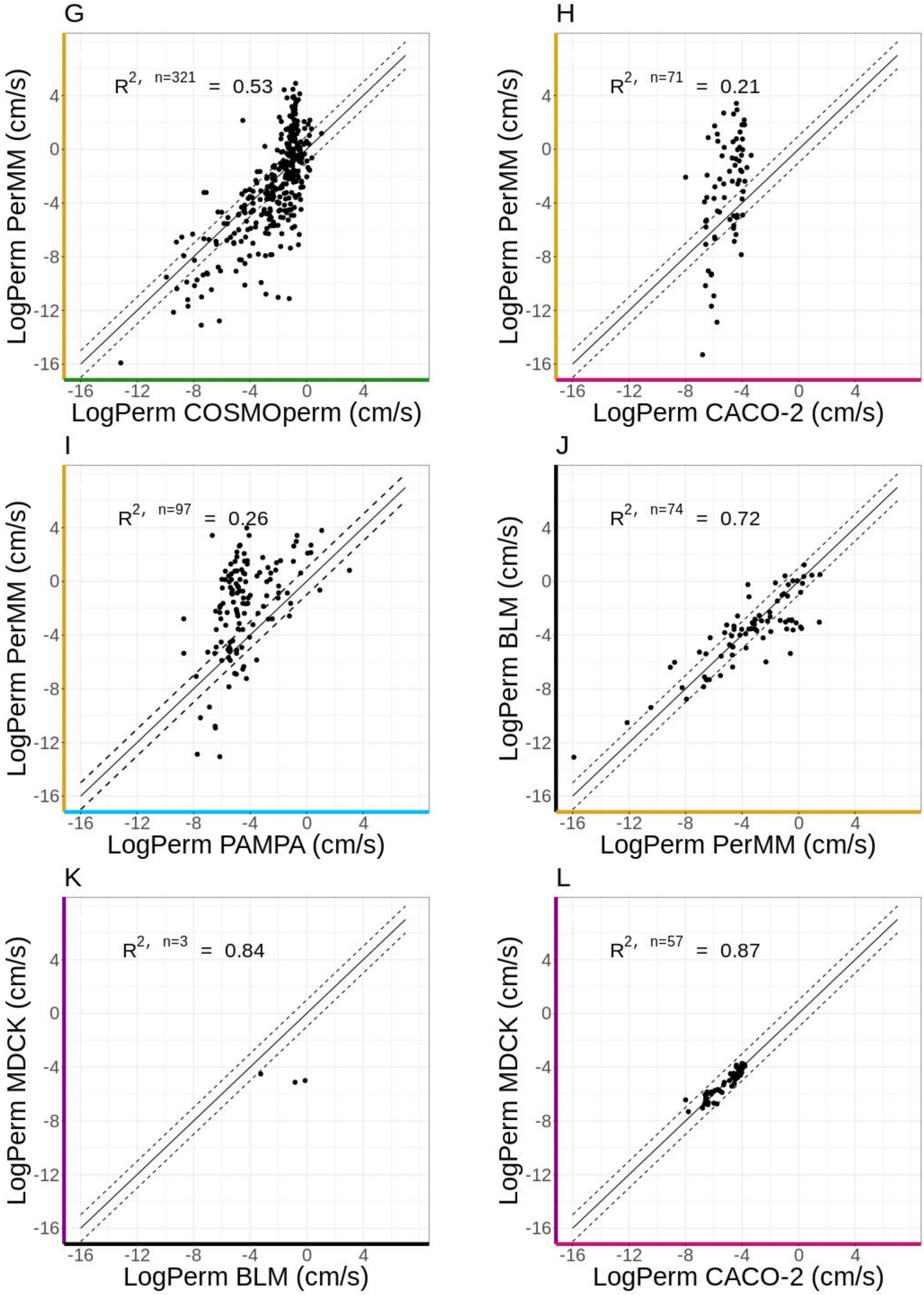

G
R^2, n=321 = 0.53
LogPerm PerMM (cm/s)
LogPerm COSMOperm (cm/s)
H
R^2, n=71 = 0.21
LogPerm PerMM (cm/s)
LogPerm CACO-2 (cm/s)
I
R^2, n=97 = 0.26
LogPerm PerMM (cm/s)
LogPerm PAMPA (cm/s)
J
R^2, n=74 = 0.72
LogPerm BLM (cm/s)
LogPerm PerMM (cm/s)
K
R^2, n=3 = 0.84
LogPerm MDCK (cm/s)
LogPerm BLM (cm/s)
L
R^2, n=57 = 0.87
LogPerm MDCK (cm/s)
LogPerm CACO-2 (cm/s)
4
0
-4
-8
-12
-16
-16
-12
-8
-4
0
4

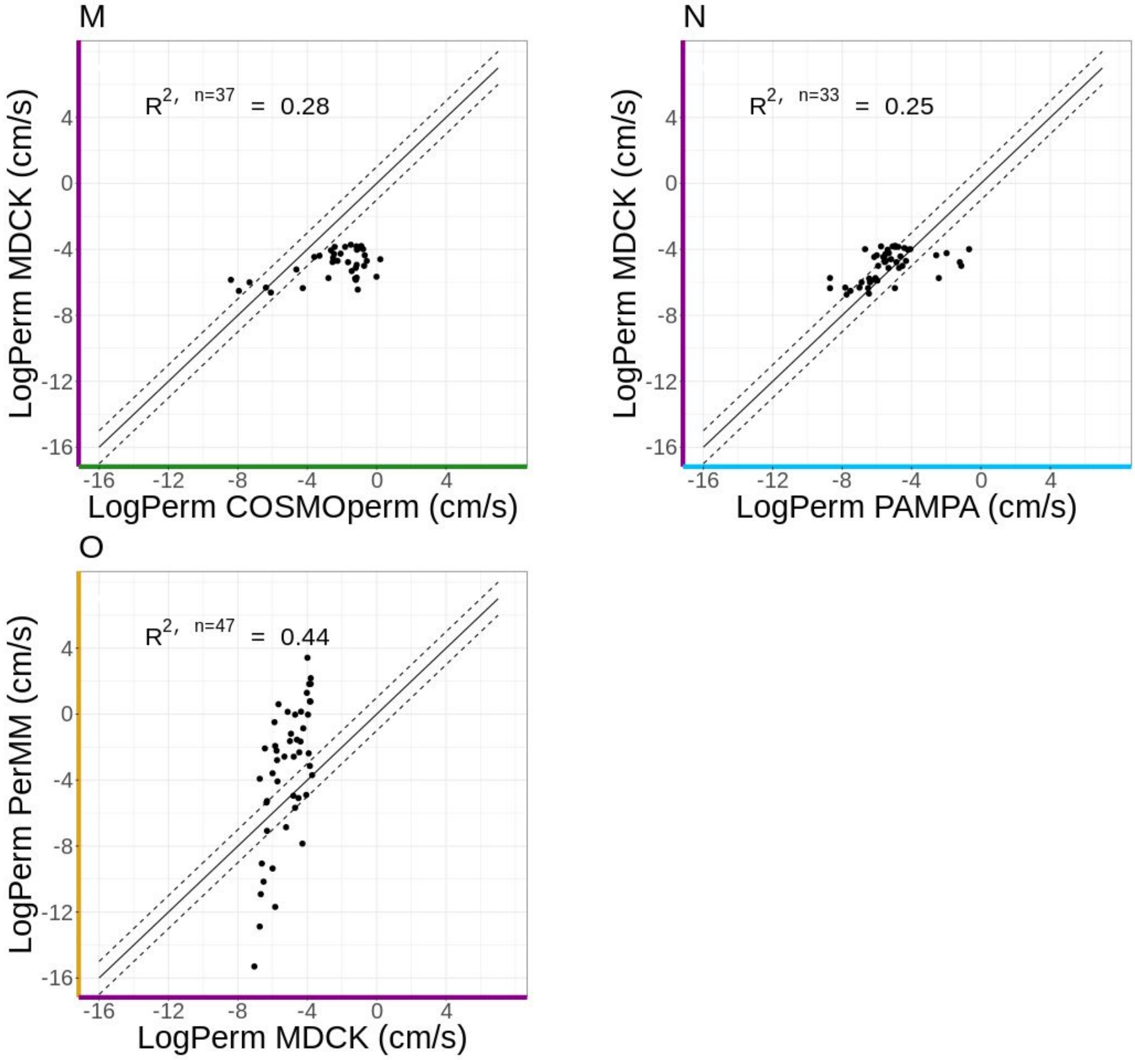
M
R2, n=37 = 0.28
LogPerm MDCK (cm/s)
LogPerm COSMOperm (cm/s)
N
R2, n=33 = 0.25
LogPerm MDCK (cm/s)
LogPerm PAMPA (cm/s)
O
R2, n=47 = 0.44
LogPerm PerMM (cm/s)
LogPerm MDCK (cm/s)
4
0
-4
-8
-12
-16
-16 -12 -8 -4 0 4

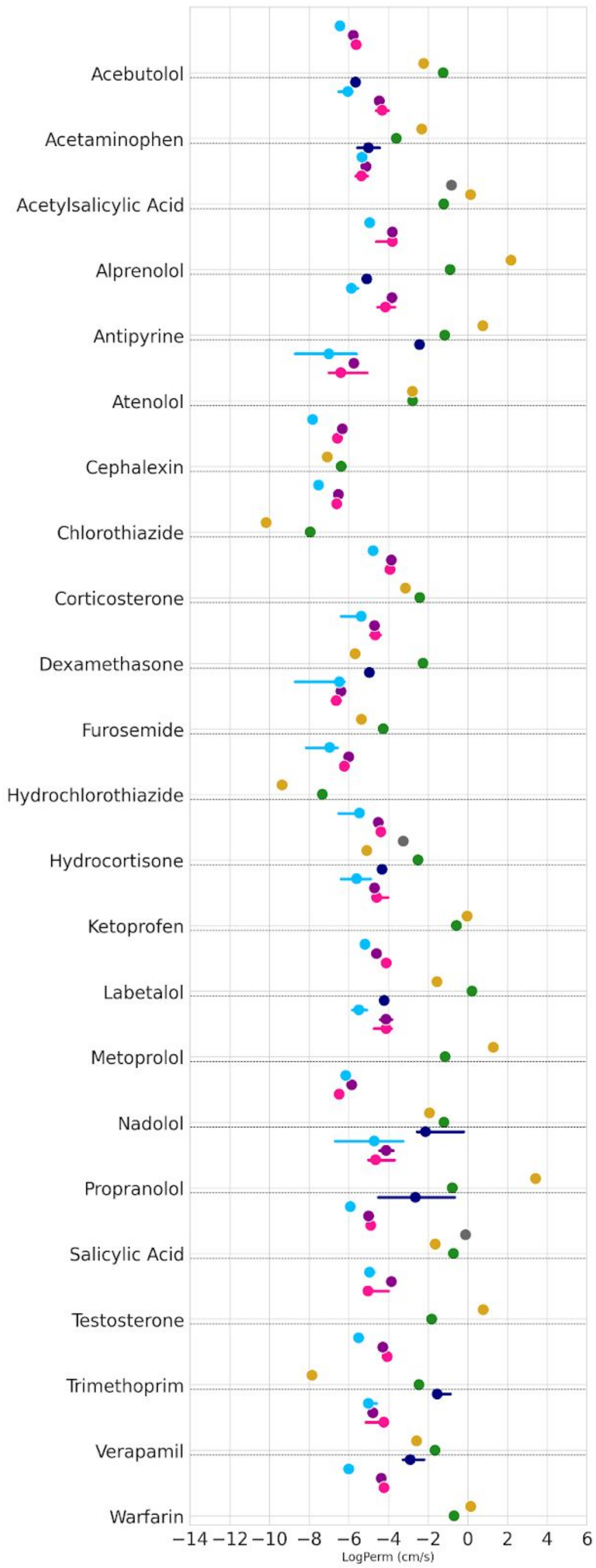

Figure S2: Intervals and medians of LogPerm values from individual methods including variability of reported data (lines) around medians (points). Coloring is the same as in the Figure 4 in the main text starting from top to down:
- PAMPA intr. (dark blue),
- PAMPA app. (light blue),
- MDCK (violet),
- CACO-2 (pink),
- BLM/liposomes (gray),
- PerMM (yellow),
- COSMOperm (green).

Table S1: Apparent permeabilities with and without UWL and with their difference, uptake ratios taken from Stenberg et al. , and logP taken from the MolMeDB database. Colours distinguish data according to logP value (green - logP < 0, white - 0 ≦ logP < 1.0, yellow - 1.0 ≦ logP < 2.0, red - logP ≧ 2.0)

**Compound**	**LogPapp (unaffected by the UWL) (cm/s)**	**LogPapp (cm/s)**	**Diff (cm/s)**	**Uptake ratio**	**LogP**
**Foscarnet**	-7.30	-7.34	-0.04	0.65	-0.16
**Amiloride**	-6.11	-6.25	-0.14	0.83	-1.08
**Mannitol**	-6.72	-6.96	-0.24	0.89	-3.59
**Raffinose**	-7.33	-7.29	0.04	1.2	-7.57
**Lactulose**	-6.57	-6.52	0.05	1.4	-5.4
**lef553**	-7.64	-7.43	0.21	0.41	-0.26
**SB217242**	-4.15	-4.26	-0.11	n/a	-
**Atenolol**	-6	-6.23	-0.23	≈1	0.45
**Sulpiride**	-6.41	-6.64	-0.23	0.87	0.56
**Cimetidine**	-5.92	-5.91	0.01	1.8	0.6
**SB209670**	-5.06	-5.01	0.05	n/a	-
**TAPP**	-7.66	-7.4	0.26	n/a	0.71
**Ciprofloxacin**	-5.72	-5.77	-0.05	0.44	1.58
**Metoprolol**	-4.04	-4.19	-0.15	≈1	1.61
**Pindolol**	-4.26	-4.44	-0.18	≈1	1.91
**Hydrocortisone**	-4.38	-4.57	-0.19	0.56	1.78
**Sildenafil**	-4.06	-4.32	-0.26	0.83	1.61
**Practolol**	-5.46	-5.74	-0.28	≈1	1.38
**Oxprenolol**	**-3.92**	-4.21	-0.29	≈1	1.99
**Antipyrine**	**-3.67**	-4.11	-0.44	1.1	1.48
**Alfentanil**	**-3.51**	-4.08	-0.57	1.1	1.38
**Oxazepam**	**-3.61**	-4.04	-0.43	1.1	2.45
**Alprenolol**	**-3.62**	-4.06	-0.44	≈1	2.15
**Nordazepam**	**-3.51**	-4.02	-0.51	1.0	3.13
**Diazepam**	**-3.12**	**-3.92**	-0.8	1.7	3.15
**Metolazone**	-5.21	-5.21	0.0	0.64	2.71
**Sulfasalazine**	-6.80	-6.80	0.0	0.087	3.7

From Table S1, it can be seen that logPerm values faster than -4 occur for molecules where logP is greater than +1. It can also be seen that the difference between logPerm values is smaller for polar molecules than for the lipophilic molecules. Metolazone and Sulfasalazine are lipophilic, and their diff is 0, but their uptake ratios suggest that they use transport pathways other than passive permeability.

In conclusion, lipophilic molecules are generally more affected by UWL than polar molecules. This rule seems to apply to molecules predominantly transported by passive diffusion.

Reference:

Stenberg, P.; Norinder, U.; Luthman, K.; Artursson, P. Experimental and Computational Screening Models for the Prediction of Intestinal Drug Absorption. *J. Med. Chem.* **2001**, *44* (12), 1927–1937. https://doi.org/10.1021/jm001101a.
